# Anorexia Nervosa in Polish Children and Adolescents in the Context of the COVID-19 Pandemic—An Observational Single Centre Study

**DOI:** 10.3390/nu16234112

**Published:** 2024-11-28

**Authors:** Katarzyna Anna Dyląg, Kamil Paweł Skowron, Magdalena Kurnik-Łucka, Łukasz Drzewiecki, Katarzyna Przybyszewska, Magdalena Król-Dykas, Paulina Dumnicka, Zuzanna Gawlik, Jakub Gawlik, Sebastian Sikora, Krzysztof Gil

**Affiliations:** 1 Department of Pathophysiology, Jagiellonian University Medical College, 31-121 Krakow, Poland; kamil.skowron@uj.edu.pl (K.P.S.); magdalena.kurnik@uj.edu.pl (M.K.-Ł.); magdalena.krol@doctoral.uj.edu.pl (M.K.-D.); krzysztof.m.gil@uj.edu.pl (K.G.); 2 St. Louis Children Hospital, 31-503 Krakow, Poland; lukasz.drzewiecki@dzieciecyszpital.pl (Ł.D.); katarzyna.przybyszewska@dzieciecyszpital.pl (K.P.); 3 Department of Medical Biochemistry, Jagiellonian University Medical College, 31-034 Krakow, Poland; paulina.dumnicka@uj.edu.pl; 4 Hospital of the Brothers Hospitallers of Saint John of God, 31-061 Krakow, Poland; zuzanna.nowak@alumni.uj.edu.pl; 5 University Hospital, 30-688 Krakow, Poland; jkgawlik@gmail.com; 6 SWPS University, 31-864 Krakow, Poland; ssikora@swps.edu.pl

**Keywords:** anorexia nervosa, eating disorder, adolescents, COVID-19, SARS-CoV-2, coronavirus

## Abstract

Introduction: Anorexia nervosa (AN) is a psychiatric disorder with a high mortality rate and significant prevalence in the paediatric population. Preliminary reports during the COVID-19 pandemic suggested an increased incidence of AN among children and adolescents. The objective of this study was to analyse new cases of AN before, during, and after the pandemic, with a particular focus on the physical manifestations of the disease. Methods: This single-centre, retrospective study included new AN cases from the hospital database of a regional paediatric hospital (a reference centre for AN) between 2013 and 2023. Data analysed included the duration of the disease, body mass index (BMI), weight loss, length of hospitalisation, laboratory markers (leukopenia, anaemia, hypoproteinaemia, hyperferritinaemia, hypophosphataemia, dyslipidaemia, and hypothyroxinaemia) and clinical features of malnutrition (such as amenorrhea bradycardia, pericardial effusion, and cortical/subcortical atrophy). Results: This study was conducted in a Polish regional hospital. We analysed 228 hospitalized female patients aged 10 to 18 years diagnosed with AN, focusing on clinical characteristics, biochemical markers, and the impact of the COVID-19 pandemic. The COVID-19 pandemic was shown to have a significant impact, with longer hospitalisations observed during and after the pandemic and a lower BMI on admission post-pandemic compared to pre-pandemic. In addition, nutritional treatment became more prevalent over time while biochemical markers such as anaemia, hypothyroidism, hypophosphataemia, and dyslipidaemia were statistically more common post-pandemic. Conclusions: This study demonstrates a significant impact of the COVID-19 pandemic on the clinical course and hospitalisation patterns of paediatric patients with AN. These findings suggest that the pandemic may have exacerbated disease severity and altered treatment approaches, emphasizing the need for enhanced clinical management and follow-up strategies for AN in the paediatric population during such health crises.

## 1. Introduction

Anorexia nervosa (AN) is an eating disorder characterized by progressive weight loss, intense fear of gaining weight, and distorted perception of body weight and shape [1]. The prevalence of AN differs significantly between countries, Europe and North America reporting a significantly higher prevalence than Asia and South America [2,3]. In Europe, the prevalence of AN is estimated to be 1–4% of women [4,5]. Mortality in patients with AN is significantly higher than in the general population [6]. According to a meta-analysis of global eating disorder mortality rates conducted by Arcelus et al., the standardized mortality ratio (SMR) was 5.9 (95% CI 4.2–8.3), indicating an approximately six-fold greater risk [7]. A recent study revealed that, after 5 years of follow-up, the SMR of patients with anorexia nervosa and severe nutritional problems was 15.9 [8]. The course of relapsing-remitting disease is observed among most patients [9,10]. AN occurs among children, adolescents, and adults, and a gradual decrease in the age of the first onset of the disease has been observed [11,12]. AN symptoms include limiting of energy consumption in relation to needs, resulting in a markedly reduced body weight considering age and sex, developmental trajectory and physical well-being [13]. In DSM-5, the severity of anorexia nervosa is classified into four stages according to the individual’s BMI: extreme (BMI < 15 kg/m^2^), severe (BMI 15–15.99 kg/m^2^), moderate (BMI 16–16.99 kg/m^2^) and mild (BMI ≥ 17 kg/m^2^) [6]. Furthermore, the DSM-5 classification distinguishes a subtype of anorexia nervosa, atypical anorexia nervosa, which is diagnosed when all criteria for anorexia nervosa are met, except that, despite significant weight loss, the individual’s weight is within or above the normal range. Bozzola et al. in 2024 summerised the most important risk factors and comorbidities such as persistent functional abdominal discomfort or autoimmune/autoinflammatory disorders. Furthermore, evidence indicates the influence of genetic predisposition, along with brain structure and pathways, on the development of anorexia nervosa. Finally, the gastrointestinal microbiota has also been identified as a potential risk factor for the development of anorexia nervosa due to intricate direct and indirect interactions between the gut and the brain [14].

The first reports suggesting that the COVID-19 pandemic contributed to a significant increase in cases of anorexia nervosa in the pediatric age group were published in July 2020 [15]. The authors suggested that the circumstances created by the pandemic caused an increase in both morbidity and severity of AN [16]. Since then, it has been well established that the COVID-19 pandemic affected both patients with pre-existing eating disorders, causing an increase in severity and incidence of the symptoms [17,18] differences in the disease characteristics [19,20] as well as a worsening of general well-being [21]. Furthermore, an increased comorbidity of psychiatric disorders was observed among individuals with AN. Most of the authors concentrated on the mental health of AN patients in the context of a pandemic. However, some studies analyzed the pediatric population in terms of increased occurrence of new cases of AN and physical determinants of health in patients with AN who required hospital treatment.

Unlike most European countries, Poland was not badly affected by the first wave of pandemic regarding COVID-19 morbidity and mortality due to the early introduction of lockdown [22,23]. However, the emotional impact of severe restrictions was high [24,25,26]. The second and third pandemic waves brought a rapid and tragic increase in COVID-19 mortality [22]. Furthermore, the Polish mental health system, including child and adolescent psychiatry services, is believed to have been inefficient for years [27,28,29,30]. In the time of the COVID-19 pandemic, access to psychiatric and psychological services in Poland, as in other European countries, was limited [27,30,31], yet an increased incidence of mental health services was observed [27,30,32]. Although it is known that the COVID-19 pandemic influenced body image, self-esteem, and predisposition to eating disorders among Polish women [33] the influence of the pandemic on the incidence of AN in children and adolescents in Poland has not yet been studied.

The objective of this study was to investigate the trend of morbidity in AN before, during and after the COVID-19 pandemic and to assess the severity of malnutrition and the presence of other biomarkers of malnutrition in AN in these groups.

## 2. Materials and Methods

### 2.1. Study Design

The study was a retrospective analysis conducted at St. Louis Children’s Hospital, ul. Strzelecka 2, 31-503 Krakow, Poland, using data extracted from medical records of 228 patients hospitalised over a 10-year period between 2013–2023 that were included in the study. The inclusion criteria were a newly made diagnosis of anorexia nervosa (coded F50.00 in the ICD-10 classification) in a patient treated in an inpatient service and of female sex. The exclusion criteria were other causes of malnutrition (i.e., comorbid gastrointestinal disorder) and male sex. During the study group selection process, 17 male patients with an AN diagnosis (corresponding to 6.9% of all AN cases) and were excluded from further analysis. Anorexia nervosa affects only a very small percentage of adolescent males, and the clinical presentation of eating disorders in males may differ from that in females, often involving less restrictive methods of weight control and a focus on muscle gain rather than weight loss [34]. In our study, we included only female cases to increase the homogeneity of the study population, as the number of male cases was too small for comparative analysis and would only lead to biased results. The mean age of the participants was 13.9 ± 1.6. The duration of the disease, the body mass index (BMI) on admission, the extent of weight loss, the length of hospitalisation, and laboratory biomarkers of malnutrition (leucopenia, anaemia, hypoproteinaemia, hyperferritinaemia, hypophosphataemia, dyslipidaemia, and hypothyroxinaemia) as well as clinical ones (bradycardia, pericardial effusion, and cortical/subcortical atrophy) were analysed. The study population was divided into three study groups: before, during, and after the SARS-CoV-2 pandemic (pre-COVID, peri-COVID, and post-COVID, respectively). The epidemic state in Poland, introduced by the Ministry of Health and lasting from 20 March 2020 to 15 May 2022, was taken as the duration of the pandemic.

### 2.2. Statistical Analysis

Statistical analyses were performed using IBM SPSS. Quantitative data were shown as mean and standard deviation (SD) or median (interquartile range), depending on the distribution (normal or different from normal in the Shapiro–Wilk test). Qualitative data were presented as the number of patients in a category and the percentage of the respective group, with the exclusion of patients with a lack of data. A one-way ANOVA was selected to compare the means of normally distributed continuous variables across the three groups of patients admitted before, during, and after the COVID-19 pandemic. The Kruskal–Wallis Test was employed for non-normally distributed continuous data, providing a non-parametric alternative to ANOVA. Pearson’s Chi-Square Test was used for categorical variables, such as patient categories or demographic factors, to test for associations or differences in proportions across the groups. A paired *t*-test was used for comparing the means of dependent populations, such as on admission and at discharge, within the same group of patients, provided the data were normally distributed. Wilcoxon Signed Rank Test served as a non-parametric alternative to the paired *t*-test. A *p*-value less than 0.05 was considered statistically significant. Data integrity was ensured through a rigorous cleaning and validation process. All datasets were reviewed for accuracy, with duplicate records and incomplete entries excluded to prevent bias. All steps of data handling and analysis were documented for reproducibility, and statistical assumptions were checked to ensure valid results.

### 2.3. Ethical Considerations

The study was approved by the Ethics Committee of the Regional Board of Physicians with the approval number of OIL/KBL/15/2024 (6 May 2024). Patient confidentiality was maintained by data pseudonymization. Each patient was assigned a unique, randomly generated identification number to replace any personally identifiable information. Direct identifiers, such as names, personal ID numbers, and contact information, were removed from the dataset. Once the data needed for the analysis were collected, the information linking patient identifiers to their original identifiers was stored in an encrypted database, accessible only to the principal investigator for data verification. This process ensured compliance with relevant ethical and regulatory standards.

## 3. Results

### 3.1. General Characteristics

**Figure 1:**
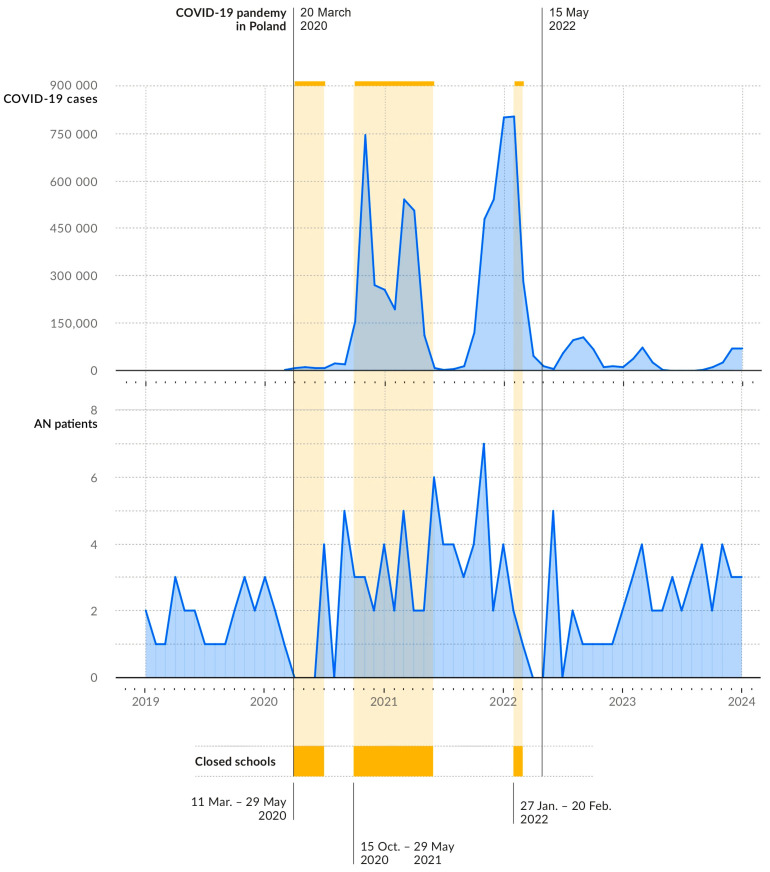
Number of cases of anorexia nervosa on the timeline juxtaposed with COVID-19 morbidity.

**Figure 2:**
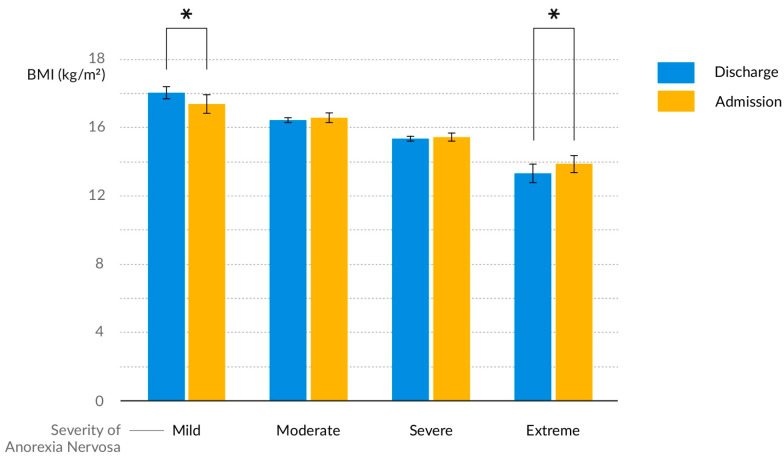
Quantitative division of the study population into disease severity categories based on DMS-V criteria based on BMI values and change in BMI during hospitalisation in each category. * *p* < 0.05.

The study included 228 hospitalised female patients diagnosed with anorexia nervosa, with an age range of 10 to 18 years. In Figure 1 we present a distribution of cases over time. Most of those included in the study exhibited a severe form of the disease, as defined by the BMI criteria outlined in the fifth edition of the Diagnostic and Statistical Manual of Mental Health (DSM-5) (Figure 2). BMI values increased slightly during hospitalisation, with a mean difference between BMI at discharge and admission of 0.457 ± 0.813 (*p* < 0.001). There was a negative correlation between BMI and ferritin levels and patients with a lower BMI at admission and a higher ferritin exhibited a longer hospitalisation period (r = −0.437 and r = 0.333, respectively, *p* < 0.001). The duration of the disease showed a positive correlation with the duration of amenorrhoea (r = 0.318, *p* < 0.001). Among biochemical indicators of the severity of malnutrition, hyperferritinaemia and hypothyroidism were the most commonly observed (Table 1). Younger age was associated with a higher prevalence of hypophosphataemia (r = −0.366, *p* < 0.001).

### 3.2. Trends Across the COVID Timeline

**Figure 3:**
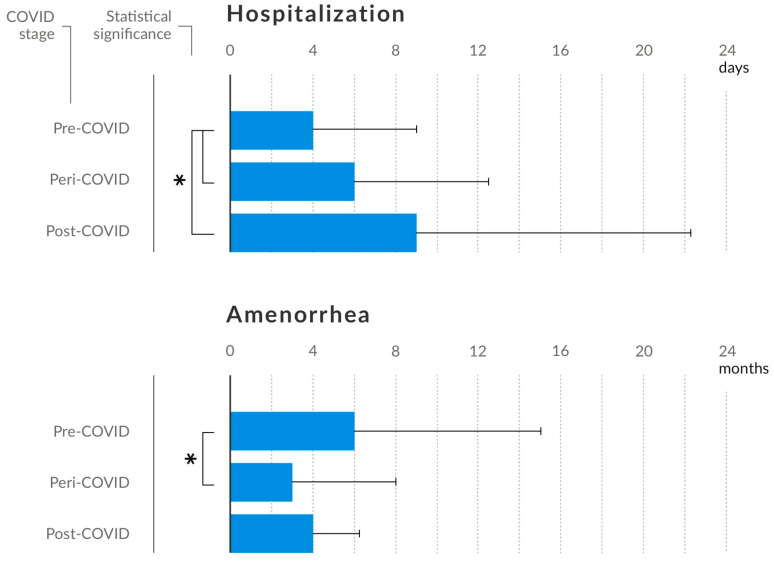
Duration of hospitalisation in days and duration of amenorrhoea in months reported on the day of admission. * Statistically significant difference between variables (*p* < 0.05).

The analysis included a population divided into three study groups: pre-COVID, peri-COVID, and post-COVID. The groups were homogeneous with respect to age, duration of the disease, reported weight loss during the course of the disease, and BMI achieved at discharge. During and after the pandemic, it was found that the duration of hospitalisation was longer than that observed prior to the pandemic (*p* < 0.001, Figure 3). In the cohort studied before the advent of the pandemic, the duration of amenorrhoea was significantly longer than in the peri-COVID group (*p* = 0.015). The BMI values on admission were comparable during and after the pandemic, although they were lower after the pandemic compared to before (*p* = 0.042).

**Figure 4:**
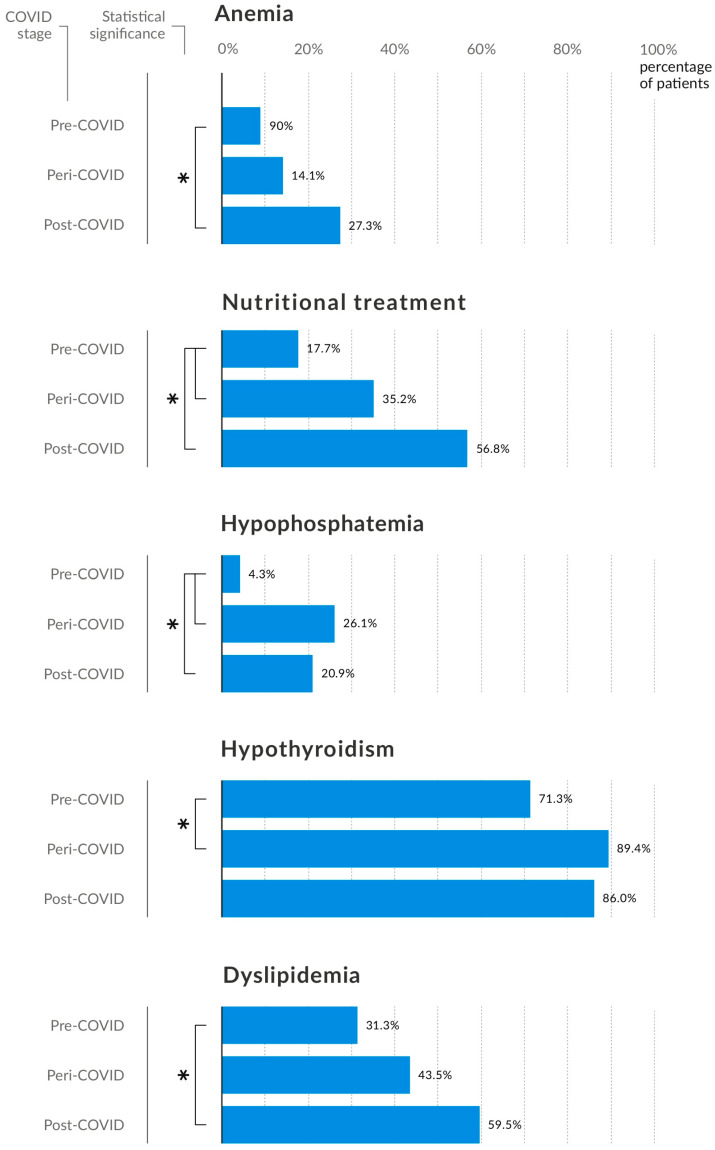
Selected characteristics that demonstrate the major differences between the study groups. * Statistically significant difference between variables (*p* < 0.05).

However, the prevalence of anaemia, decreased thyroid hormone levels, hypophosphataemia, and dyslipidaemia was higher among patients after COVID than in the pre-COVID group. Patients during the pandemic exhibited a transition period of change, as indicated by the aforementioned characteristics. Only with regard to nutritional therapy and the incidence of hypophosphataemia were there discernible statistical differences when comparing these patients with pre-pandemic patients. The percentage of patients who received nutritional treatment gradually increased from the pre-pandemic period to the post-pandemic period (Figure 4). Leukocyte, platelet, and ferritin levels were found to be comparable between the study groups. However, the prevalence of anaemia, decreased thyroid hormone levels, hypophosphataemia, and dyslipidaemia was higher among patients after COVID than in the pre-COVID group. Patients during the pandemic exhibited a transition period of change, as indicated by the aforementioned characteristics. Only with regards to nutritional therapy and the incidence of hypophosphataemia were there discernible statistical differences when comparing these patients with pre-pandemic patients.

**Figure 5:**
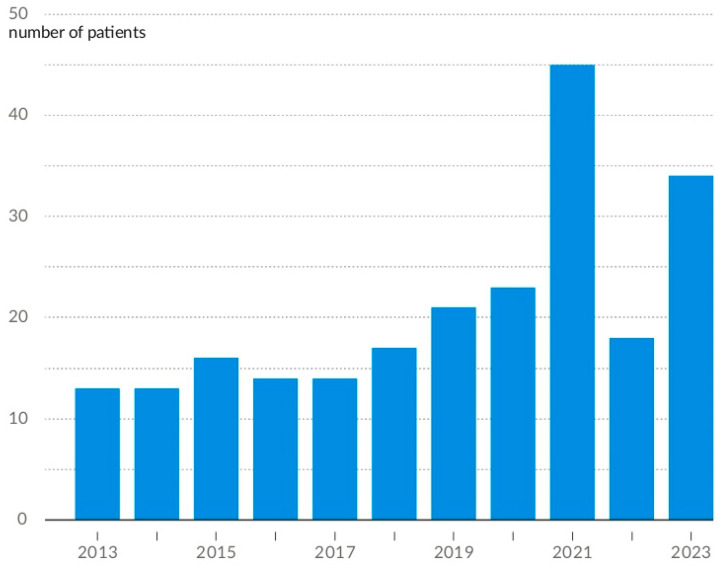
Annual number of AN hospitalisations from 2013 to 2023.

**Figure 6:**
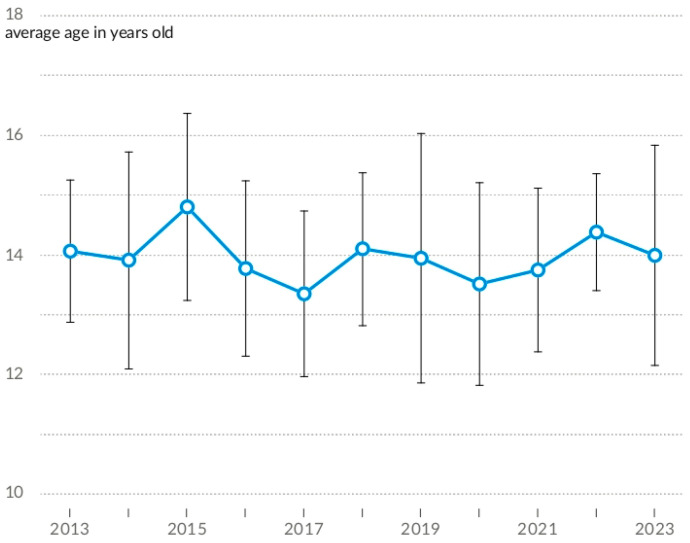
Annual average age of AN patients hospitalised between 2013–2023.

Data on hospitalisations on an annual basis reveal a notable increase in 2021, followed by a precipitous decline in the following year. In 2023, the upward trajectory observed during the previous decade was resumed. Annual trends in AN hospitalisations are presented in Figure 5. The age distribution of hospitalised patients has remained relatively stable over time (Figure 6).

## 4. Discussion

In our study, we observed a rapid increase in hospitalizations due to anorexia nervosa during the COVID-19 pandemic with an increase in hospitalization duration, nutritional treatment requirement, and duration of amenorrhea. This result is consistent with reports from pediatric centers in other countries. Haripersad et al. [15] and Hansen were the first to alarm the medical community about the increase in new hospitalisations due to AN during the first lockdown; however, due to the time when they were published, their reports were lacking more in-depth analysis. Lin et al. [35] established a significant increase in hospital admissions due to AN accompanied by an extension of hospitalisation. Otto et al. [36] reported the same trend, which observed a double incidence in AN during COVID-19 compared to before COVID-19 years. Matthews et al. [37] suggested that the odds of hospitalization for patients with AN were eight times higher during the pandemic than in previous years. Agostino et al. [38] established that not only the incidence of AN increased with pandemic, but also patients diagnosed with AN before pandemic were characterized with fewer markers of disease severity than patients diagnosed during pandemic. Similarly, Vyver et al. [39] demonstrated that AN morbidity doubled during the COVID-19 period. Girardi et al. [40], Hurtado et al. [41], Rafferty at al. [42], Silber et al. [43], Hyam et al. [44], Wong et al. [45] and Giraldo et al. [46] documented an increased AN-related admission rate in their respective countries. Schlapfer et al. showed that an increase in admissions due to AN was associated with a prolonged admission time and nutritional interventions. Goldberg et al. [47] concluded that the increase in admissions was not accompanied by an increase in the severity of the disease. In 2023 Schlissel et al. presented a review in which they observed during COVID-19 pandemia increased incidence of AN and AAN among adolescents. They concluded that overall the pandemic has exacerbated symptoms specific to eating disorders, hence straining an already overextended healthcare system [18]. Increases were also observed in other non-Western nations, including Pakistan and Singapore, but in these cases, investigators did not distinguish between forms of eating disorders [48,49]. To date, there have been no Polish data on trends in new admissions and the severity of AN in light of the pandemic. However, our findings confirm general trends throughout the world [15,18,20,36,37,38,39,40,41,42,43,44,47,50,51,52]. To our knowledge, this is also the first study to analyze post-pandemic trends in the incidence of AN. In our sample, we observed that the trend of increased morbidity from AN continues in the years after COVID-19. Hurtado et al. analyzed peripandemic and postpandemic years together and their observations are consistent with ours [41], however, this trend should be carefully researched in the near future. Interestingly, Herpetz-Dahlman et al. indicated that after pandemic admission rates due to AN remained high among children with a decrease among adolescents [53]. The trend should be carefully studied in the future.

Anorexia nervosa is a psychiatric condition with a significant impact on the biological mechanisms of the human body [54]. In our peri-pandemic and post-pandemic cohorts, we report a higher frequency of anemia, hypothyroidism, hypophosphatemia, and dyslipidemia compared to the pre-pandemic cohort. These parameters offer a more precise reflection of patients’ somatic state, thereby providing a more nuanced understanding of their physical condition that extends beyond the limitations of a BMI-based approach to disease severity assessment [55]. One way of gaining a deeper insight is to monitor the negative effects of malnutrition on the functioning of the endocrine system, causing disorders of the hypothalamic-pituitary-adrenal, gonadal or thyroid axis that can mostly be interpreted as response reaction to systemic illness which may also simultaneously serve as adaptive mechanisms. For instance, it appears that the reduced levels of thyroid hormones in individuals with anorexia nervosa have an adaptive role in response to malnutrition to slow down metabolism and thus reduce the body’s energy expenditure (resting energy expenditure) [56]. It is often accompanied by increases in serum ACTH and cortisol levels (maintaining euglycemia) and decreases in serum gonadotropin and sex hormone concentrations (reducing energy expenditure), as observed in malnourished patients. Hypothyroidism, together with hypoinsulinemia, increased endogenous cholesterol synthesis and elevated cortisol levels, may contribute to dyslipidaemia. Additionally, severe malnutrition and metabolic disorders can result in anaemia, which is primarily caused by bone marrow hypoplasia. It can thus be inferred that the elevated prevalence of the aforementioned disorders observed in the peri- and/or post-COVID-19 patient population indicates a more severe somatic presentation of the AN than was observed in the pre-pandemic period, despite only slight differences in BMI between those groups. Few researchers have investigated the influence of pandemic on malnutrition biomarkers in patients with AN. Stra [57] face et al. observed that hospitalized patients had leukocytopenia, neutropenia, hypovitaminosis, and hormonal disturbances; however, the authors only illustrated the COVID-19 cohort without comparing it with the prepandemic cohort. Hurtado et al. included sex hormones and vitamin B12 levels in the analysis, but no differences were observed between the two analyzed groups analyzed [41]. Goldberg et al. reported a higher incidence of leucopenia among patients hospitalised during the pandemic, but did not confirm the differences in terms of hypophosphatemia and thyroid hormones levels [47]. Both pre-COVID-19 and post-COVID-19 AN patients were similar in laboratory malnutrition parameters according to Girardi et al. [40]. However, none of the authors reported differences in BMI between the groups. These findings raise a question about methods to estimate the severity of the disease in AN.

The disease severity criteria commonly used in DSM-5, which are based on patient BMI values, are increasingly being criticized for their lack of real-world applicability in terms of translating into the somatic or psychological state of patients [58,59]. In relation to this, efforts are being made to identify a new, more clinically useful indicator of disease severity. From a psychopathological point of view, methods that assess the severity of the cardinal characteristics of AN, such as the drive for thinness and overestimation of weight and shape, are being used, which are gaining increasing support in the scientific literature [60,61]. However, similar to the assessment of BMI, these methods do not provide a reliable reflection of the biological manifestations of the disease such as endocrine or electrolyte disturbances, which also directly influence therapeutic decisions. This is particularly relevant given the considerable prevalence of systemic complications in this population, including anemia, hyponatraemia and hypokalaemia, or elevated liver enzymes, which cannot be directly attributed only to the degree of undernutrition [62]. However, new reports are periodically published on potential new indicators of disease severity. One such example is a report which suggests that the evaluation of a patient’s body weight history in relation to their body weight upon admission is associated with nutritional biomarkers and can be a useful method for classifying the severity of disease [63].

There are various interpretations of the reasons behind the increase in admissions due to new AN during the pandemic. Sociocultural factors are of great importance in body image and are known to be essential determinants of the development of anorexia nervosa [64,65,66] In fact, stress of any kind should be considered as a precipitating factor for the onset of AN [65]. Our study specifically identifies social isolation related to the COVID-19 pandemic as another important factor in the development and aggravation of AN. During the COVID-19 pandemic, educational institutions were closed and most extracurricular activities for children and adolescents that usually take place outside the family and in group settings were canceled. Teens experienced an extended period of physical isolation from their peers, teachers, extended family, and community connections. Social distancing and school closures exacerbate mental health issues of children and adolescents, who are already more susceptible to such problems than adults [67]. In addition, quarantine experiences were often associated with decreased psychological well-being and the emergence of psychological symptoms and emotional disorders, including sadness, anxiety, sleeplessness, and post-traumatic symptoms [68]. It should be mentioned that Graell et al. reported that people with a history of eating disorders have been among the most severely affected [69]. Moreover, 41% of adolescents receiving therapeutic care exhibited a resurgence of eating disorder symptoms following the lockdown, especially among those with diminished self-directedness and fewer effective coping mechanisms [70]. Furthermore, lock-down and isolation due to COVID-19 pandemic increased the use of internet and social-media, which are already known to affect loneliness [71,72]. Women, in particular, rely on social networks, which can negatively influence their perceptions of body image [57,58]. However, the long-lasting consequences of social restraint for newly diagnosed and relapsing patients with AN are unknown. Moreover, the concept of control has been considered a crucial factor in the development of AN. It has been established that people with AN tend to control their caloric intake in response to the inability to control the uncertain aspects of life [73,74]. It is difficult to imagine something more uncontrolled than the rapidly developing worldwide pandemic and the sense of instability can be considered a precipitating factor in many of the new cases of AN. Jarvers et al. documented that loss of personal control and alexithymia contributed to the symptomatology of AN during the pandemic [75].

Furthermore, the fact that medical institutions provided care predominantly by telehealth methods seems to be an important factor. Telehealth, being an effective tool that helps provide better care to patients, has several limitations, especially in terms of surveillance of patients with mental health conditions [76]. Therefore, AN diagnoses may have been delayed, resulting in a more severe clinical picture. Patients with already diagnosed AN reported dissatisfaction with telehealth services [77] and a decrease in treatment adherence was observed [78], which might suggest that in this particular group direct communication with physicians and therapists could be crucial [79]. Additionally, children and adolescents were home schooled during lockdowns. Teachers and school psychologists could not serve as whistleblowers in the context of individual weight loss. Furthermore, the crucial role of family structure in the pathophysiology of AN is undisputable [80,81]. During lockdowns, all family members were closed in a limited space, which could have increased the impact of the family and increased the outbreak of the disease. There are various interpretations of the reasons for the increase in AN cases observed during the pandemic. Most interpretations take into account psychosocial factors. It should be mentioned that direct biological mechanisms that lead to an increase in susceptibility to AN cannot be excluded on the basis of current scientific data. However, if such trends of AN related to post-COVID morbidity are maintained, especially in light of the continuously unstable epidemiological situation, such mechanisms must be searched for.

According to the latest report of the World Health Organization, Polish children have one of the lowest rates of mental well-being and one of the highest rates of attempted suicide in Europe [82]. Our results further demonstrate such a defect in the Polish healthcare system in the context of pediatric mental health and AN, which has only been aggravated by the COVID-19 pandemic. On the other hand, such pandemic-related unpreparedness was not uncommon [15,36,39,40,41,42,50,51,52,79,83,84], which could argue for the intersection between biological and psychological mechanisms related to SARS-CoV-2 infection [85]. Thus, our results also imply the global need for dedicated health policies, which could be easily implemented into virtual (e.g., telehealth) clinical practice in the time of possible future pandemics, military conflicts or climate-related migration and disasters. These strategies should be established for a better and earlier diagnosis of AN, among other psychiatric disorders, to prevent hospitalization. The multilevel prevention system to support youth mental well-being should be introduced across different health promotion settings, including the implementation of feasible lifestyle recommendations combined with practical interventions. Special attention should be paid to up-to-date sleep and nutritional recommendations. The latest research suggests causative links between sleep duration, screen time and mental health [86]. And the bidirectional relationship between diet quality and mental health in children and adolescents has also been highlighted [87,88].

The main strength of our study is its comprehensive approach and especially the analysis of the biological aspects of AN in the context of a pandemic. Furthermore, we included the post-pandemic perspective in the analysis. However, several limitations of our study must be acknowledged. This is a single-center retrospective study that may contribute to some selection bias. However, in the region, St. Louis Children’s Hospital is the only service that provides pediatric and psychiatric services, and the vast majority of cases of AN are hospitalized at this facility. Only patients who required hospitalization were included in the study; What can limit conclusions about the trend of morbidity to more serious cases, the overall increase in cases of AN remains unknown. We also limited our analysis to only female patients. This decision was motivated by the fact that the characteristics of AN are more homogeneous among women. Despite limitations, the study offers a novel approach in analyzing trends among AN patients in the context of a pandemic. Further research is needed to fill the gaps in knowledge on interpretations of trends in the post-pandemic context. Our study also advocates for well-planned and AN-dedicated countermeasures in the event of future pandemics [89], which are believed to be inevitable [90], and the social restrictions associated with them.

## 5. Conclusions

The study provides an insight into the trajectory of AN in the context of the COVID-19 pandemic, and, as previously documented by other researchers, it highlights a notable increase in the number of AN cases during the SARS-CoV-2 pandemic. However, an extremely important finding seems to be that the pandemic not only increased the incidence (or manifestation) of AN, but also worsened its clinical presentation, as evidenced by longer hospital stays, an increased need for nutritional interventions, and higher rates of anaemia, hypothyroidism, hypophosphataemia, and dyslipidaemia after the pandemic. The observed changes in these parameters, which may be regarded as malnutrition-related biomarkers, suggest that the disease severity in the peri-pandemic period is greater despite the presence of subtle differences in BMI values. Given the reported trends, it is crucial to consider the potential long-term effects of the pandemic on mental health, particularly in vulnerable populations such as adolescents. The study underscores the need for enhanced awareness, early intervention, and targeted support strategies to mitigate the impact of future pandemics on eating disorders like AN.

## Figures and Tables

**Table 1:** General characteristics of the study groups. WBC, white blood cells; PLT, platelets. Bold—statistically significant difference between groups. Data are presented as mean ± SD or median (interquartile range).

Variable	All Patients (*n* = 228)	Pre-COVID (*n* = 113)	Peri-COVID (*n* = 71)	Post-COVID (*n* = 44)	*p*
Age (years)	13.9 ± 1.6	14 (2)	14 (2)	14 (2)	0.531
Hospitalisation (days)	5 (8)	4 (5)	6 (6.5)	9 (13.25)	**<0.001**
Nutritional treatment	30.7%	17.7%	35.2%	56.8%	**<0.001**
Height (cm)	160 (10)	159 (12)	161 (10)	161 (9.8)	0.121
Weight at admission (kg)	35.9 ± 6.4	36.2 ± 7.3	35.9 ± 5.4	35.4 ± 5.5	0.766
BMI at admission (kg/m^2^)	14.0 ± 1.7	14.4 ± 1.9	14.1 ± 1.4	13.7 ± 1.6	**0.047**
Weight at discharge (kg)	36.6 ± 5.7	36.1 ± 6.6	36.7 ± 4.9	37.7 ± 4.9	0.324
BMI at discharge (kg/m^2^)	14.5 ± 1.4	14.5 ± 1.6	14.4 ± 1.2	14.5 ± 1.1	0.822
Amenorrhoea (months)	4 (5)	6 (9)	3 (5)	4 (2.25)	**0.018**
Duration of the disease (months)	7 (8)	6 (8)	7 (8)	6.5 (7.75)	0.816
Weight loss during the disease (kg)	11 (8)	11 (8)	11 (8.5)	10 (7.1)	0.701
WBC (×10^3^/µL)	5.22 ± 1.72	5.45 ± 1.65	5.13 ± 2.1	4.75 ± 1.1	**0.013**
PLT (×10^3^/µL)	225.5 ± 54.6	225.1 ± 54.5	223.8 ± 57.3	229.3 ± 51.5	0.918
Ferritin (µg/L)	122.3 (108.7)	110.5 (85.3)	133.4 (115.2)	122 (103.9)	0.295
Leukopenia, *n* (%)	28.8%	21.6%	38%	31.8%	0.051
Anaemia, *n* (%)	14.2%	9%	14.1%	27.3%	**0.013**
Hypoproteinaemia, *n* (%)	5.5%	5.7%	8.6%	0%	0.145
Hyperferritinaemia, *n* (%)	71.1%	71.4%	68.8%	74.4%	0.817
Hypothyroidism, *n* (%)	80.6%	71.3%	89.4%	86%	**0.011**
Pericardial fluid, *n* (%)	27%	32.8%	26%	18.9%	0.319
Bradycardia, *n* (%)	38.5%	42.3%	38.2%	29.3%	0.244
Cortical-subcortical atrophy, *n* (%)	26.1%	35.4%	19.3%	19.4%	0.082
Hypophosphataemia, *n* (%)	15%	4.3%	26.1%	20.9%	**<0.001**
Dyslipidaemia, *n* (%)	43.3%	31.6%	42.4%	61%	**0.015**

## Data Availability

The data presented in this study are available on request from the corresponding author due to privacy reasons.
